# Cursor control by Kalman filter with a non-invasive body–machine interface

**DOI:** 10.1088/1741-2560/11/5/056026

**Published:** 2014-09-22

**Authors:** Ismael Seáñez-González, Ferdinando A Mussa-Ivaldi

**Affiliations:** 1 Department of Biomedical Engineering, Northwestern University, McCormick School of Engineering and Applied Science, 2145 Sheridan Road, Evanston, IL 60208, USA; 2 Department of Physical Medicine and Rehabilitation, Northwestern University, Feinberg School of Medicine, 710 North Lake Shore Drive, #1022, Chicago, IL 60611, USA; 3 Sensory Motor and Performance Program, Rehabilitation Institute of Chicago, 345 E. Superior St, Suite 1406, Chicago, IL 60611-2654, USA

**Keywords:** body–machine interface, wheelchair, spinal cord injury, Kalman filter, cursor control, motor learning

## Abstract

**Objective:**

We describe a novel human–machine interface for the control of a two-dimensional (2D) computer cursor using four inertial measurement units (IMUs) placed on the user’s upper-body.

**Approach:**

A calibration paradigm where human subjects follow a cursor with their body as if they were controlling it with their shoulders generates a map between shoulder motions and cursor kinematics. This map is used in a Kalman filter to estimate the desired cursor coordinates from upper-body motions. We compared cursor control performance in a centre-out reaching task performed by subjects using different amounts of information from the IMUs to control the 2D cursor.

**Main results:**

Our results indicate that taking advantage of the redundancy of the signals from the IMUs improved overall performance. Our work also demonstrates the potential of non-invasive IMU-based body–machine interface systems as an alternative or complement to brain–machine interfaces for accomplishing cursor control in 2D space.

**Significance:**

The present study may serve as a platform for people with high-tetraplegia to control assistive devices such as powered wheelchairs using a joystick.

## 1. Introduction

Damage to the spinal cord causes long-lasting and devastating loss of motion, coordination, weakness, and altered reflexes, usually below the level where the injury occurred. However, even when the injuries occur at a high level of the spinal cord, some residual motor and sensory capacity remains available. These functions serve as the means to control assistive devices such as tools, computers, and wheelchairs.

There are more than 150 000 users of powered wheelchairs in the United States (Edwards and McCluskey 2010). Despite progress in the field of assistive technologies, there are still major barriers that obstruct the effective and safe use of powered wheelchairs. The possibility of encountering difficulties and accidents is significantly higher for individuals with poor control of their upper body (Hunt *et al* 2004). One of the first challenges for them is to learn how to interact with the different available interfaces and how the vehicles respond to their actions. Sip-and-puff switches, and head-and-chin devices operate the commercially available devices and their controls for people without enough arm coordination to control a joystick (Ding and Cooper 2005). Other novel methods incorporate inertial measurement units (IMUs) on the head (Mandel *et al* 2007) or electroencephalography (EEG) (Iturrate *et al* 2009, Carlson and Demiris 2012) to convert individuals’ intentions into steering commands for a powered wheelchair.

The commercially available interfaces for this population —like the sip-and puff and the head-and-chin systems—operate with a discrete directionality, meaning that the user can only move in one direction at a time from a predefined ‘vocabulary’ (right, left, front, or back). They are commonly non-proportional, which means that no matter how much pressure a user exerts on their device, the wheelchair will always move at the pre-determined speed. Moreover, these systems are obstructive to the head and mouth, so unless the users are moving in ‘locked’ mode where the wheelchair maintains a constant forward velocity, they must apply continued pressure and can’t engage in conversation or look around while they operate their vehicles.

A survey on the use of powered wheelchairs found that more than 50% of users report complaints with their wheelchair control (Fehr *et al* 2000). Forty per cent reported difficulties in steering and manoeuvring tasks, and 10% found it ‘extremely difficult or impossible’ to use their wheelchairs. Clinicians interviewed in the same study highlighted the importance of successful learning in order to overcome the barriers that limit the access to current assistive devices. However, current devices offer a fixed vocabulary of commands and the interactions are strictly constrained. This conventional approach places the burden of learning to operate the wheelchair entirely on the user.

Even in individuals with injuries to the cervical spinal cord, some motor and sensory capacities may remain available in the upper-body. While the commercially available systems do not provide a flexible approach to the user's surviving skills, researchers in brain–machine interfaces are promising the possibility to operate wheelchairs and other devices by recorded neural activities (Wolpaw and McFarland 2004, Lotte *et al* 2007, Rebsamen 2008, Chadwick *et al* 2011, Hochberg *et al* 2012). However, in so doing, the brain-machine interface does not promote the use of what remains available in terms of residual body motions. Keeping an active body is critical for people with high-tetraplegia in order to avoid collateral effects of paralysis such as muscular atrophy, chronic pain, and to recover some of the lost mobility (Levy *et al* 1990, Topka *et al* 1991, Chen *et al* 1998, Chen *et al* 2002, Hesse *et al* 2003)

To overcome these limitations, assistive devices should not follow the current ‘one-size fits all’ approach. Instead, they should be client-based (Fehr *et al* 2000). It is crucial to develop the next generation of assistive devices that continuously adapt to each individual’s residual mobility and evolving skills. For this purpose, we have developed a novel approach for a body–machine interface that harnesses the overabundant number of signals from the cache of body movements that users are still capable to execute. This allows the users to take advantage of the natural ability of the motor system to reorganize the control of movement (Chen *et al* 2002) so as to achieve a qualitatively and quantitatively greater degree of integration between body and machine that has not been possible in the past.

In this report, we describe a novel method for a body—pmachine interface that aims at allowing people with high-level paralysis to communicate their intended actions using their individual motor capacities. In an experimental setup analogous to (Paninski *et al* 2004), unimpaired subjects wore four IMUs on the shoulder area and learned to control a cursor on the screen using a Kalman decoder (Welch and Bishop 1995).

A calibration procedure where subjects were instructed to follow a smoothly moving cursor on the screen as if they were controlling it with their shoulder motions allowed us to train a Kalman filter that decoded upper-body kinematics into cursor kinematics. Our methods build on previous work in brain–-machine interfaces (Brown *et al* 1998, Wu *et al* 2002) where spike trains recorded from cortical neurons guided the motion of a cursor on a computer screen. Here, we explore the control of the cursor using a non-invasive and intuitive approach that exploits the residual mobility that remains available to the paralyzed users of assistive devices. The redundancy in our algorithm comes from using as much information from the body as possible to estimate the cursor’s control. However, more information is not always better, as adding more noisy sensor data might actually degrade performance of the decoding algorithm.

In this study we analysed the effect that adding more information in the observation vector of the decoding algorithm has on cursor control performance. We asked subjects in three different groups to perform a centre-out reaching task with a cursor controlled by movements of their shoulders. Each group had a different amount of information in the observation vector used for the calibration and decoding components of the Kalman algorithm. The first group (E) used the Euler angles of the IMUs placed on their shoulders to control the cursor. The second group (EV) used Euler angles and angular velocities. The third group (EVA) used Euler angles, angular velocities, and linear accelerations. We compared performance during the reaching task between the three different groups.

After assessing the role of multiple state components in the observation vector that the algorithm uses to drive the controlled cursor, we used the map that demonstrated the best performance for a second set of experiments. Subjects performed a reaching task where they were required to move the cursor to multiple targets in multiple positions, including trials without visual feedback of the cursor, and five additional targets that were not seen during training. We also placed subjects in a virtual wheelchair environment where subjects were instructed to perform real navigation tasks commonly used to assess wheelchair control ability (Archambault *et al* 2012).

Using an algorithm that exploits the abundance and redundancy of individuals’ residual motion might simplify the decoding problems faced by current brain–machine interfaces (Fehr *et al* 2000, Wolpaw and McFarland 2004, Lotte *et al* 2007, Chestek *et al* 2009, Kim *et al* 2011, Orsborn *et al* 2012). The current exponential decrease in IMU technology cost and size (Yole Developpement 2012) could allow users in the future to incorporate more than four sensors into the body–machine interface and increase the redundancy of the motion signals even further and potentially increase their control performance.

## 2. Methods

### 2.1. Experimental setup

**Figure 1:**
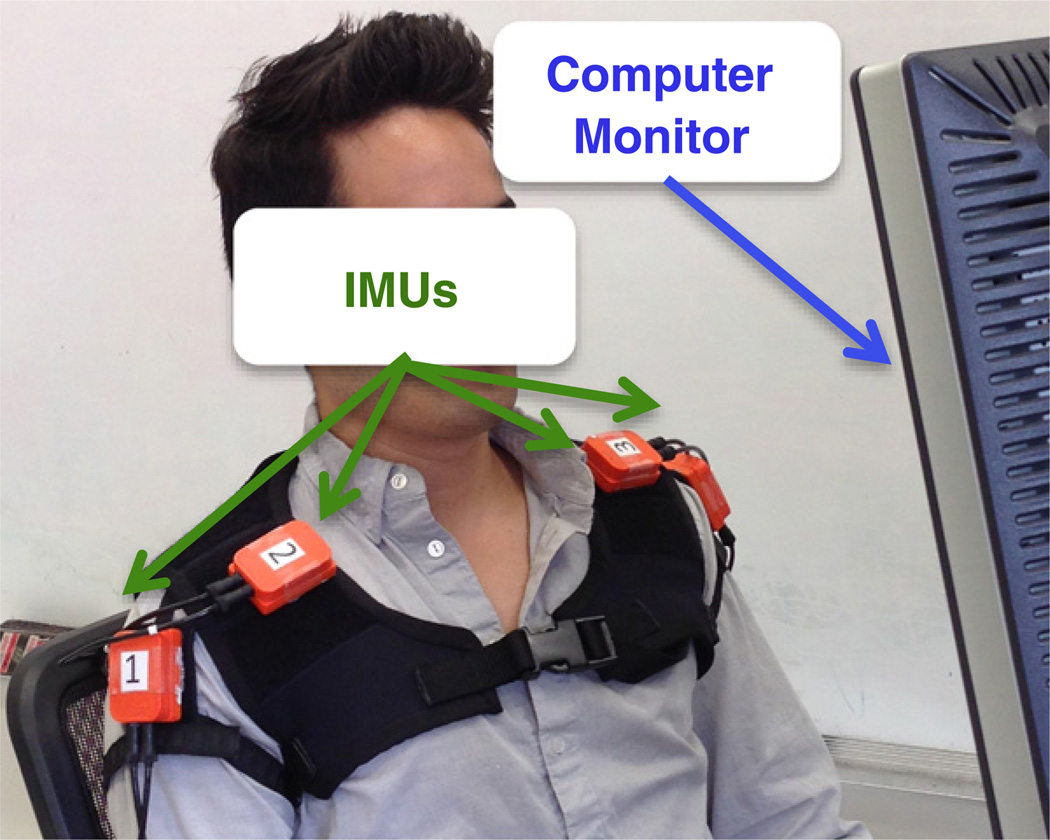
Experimental setup. The subject sits in front of a computer monitor wearing a vest with four IMUs attached to the shoulders.

Twenty-eight healthy subjects (16 female, 12 male, 24 ± 6 years old) gave their informed and signed consent to participate on this study, which was approved by Northwestern University’s Institutional Review Board. Subjects sat in front of an 18 × 18 cm computer display wearing a motion vest with Velcro® patches on the shoulder areas. Four IMUs (MTx, Xsens Technologies B.V., Enschede, Netherlands) were attached to the Velcro as shown in ‘figure 1’. The IMUs were connected to a CPU via an Xbus Master (MTx, Xsens Technologies B.V., Enschede, Netherlands) digital data bus system, and the combination of the 3D accelerometers and gyroscopes inside them allowed us to capture combinations of shoulder elevation, depression, adduction, and abduction. Data from the IMUs were sampled in real-time (Simulink, Mathworks) at a rate of 50 Hz.

### 2.2. Protocol

The main objective was to map the 24D signals from the body motions, measured by the IMUs, to the control of the 6D kinematics of the cursor on the screen. A calibration procedure provided us with this map via a Kalman filter (Welch and Bishop 1995) as applied by Wu *et al* (Wu *et al* 2002) in their brain–machine interface.

#### 2.2.1. Calibration

Subjects were presented with a cursor that moved on the monitor and were asked to move their shoulders with the cursor, as if they were controlling it. They were instructed to ‘follow’ up and down cursor movements by moving their right shoulder up and down (elevation and depression respectively), and to follow right and left cursor movements by moving their left shoulder up and down respectively.

The 1 cm diameter cursor moved through a predetermined centre-out path with a cosine velocity profile so that the cursor’s position history while moving right /left followed the function:

(1) $$x_{t}=5\text{sin}(\frac{\pi }{4}t),$$

(2) $$y_{t}=0$$

Here *t = k*d*t* with *k* = 1,… ,200 and d*t* = 0.02, so that the total duration of each cursor movement from the centre to the right direction and back lasted for a total of 4 s. A 6×6cm box enclosed the cursor’s movement range so that subjects knew when the cursor was going to reach the edge and come back to the centre, and they could plan to move their shoulders accordingly. Each of the four directions was reached six times for a total calibration time of 96 s.

Cursor and body motion data were logged during the calibration phase. The position, velocity, and acceleration of the cursor were recorded at each time step *k* (every 20 ms) as the cursor’s *state*, i.e. $s_{k}={\left[x,y,v_{x},v_{y},a_{x},a_{y}\right]}_{k}^{T}$ where s_*k*_∈ R6 × 1. The IMUs Euler angles, angular velocities, and linear accelerations were recorded as the body *observation* in a 24-dimensional vector *z_k_*= {[2-Euler angles (roll, pitch) + 2-gyroscope + 2-accelerations]*4 sensors} at each time step *k* as the subjects followed the cursor with their body. Both data were fed into a Kalman filter to learn the mapping between body motions and cursor kinematics.

#### 2.2.2. Kalman filter algorithm

The main goal of the Kalman filter is to make an estimation of the cursor’s *state*, at every instant in time. The Kalman model assumes the cursor’s state at time *k* to be linearly related to the future state at time *k* + 1 via the stochastic linear function

(3) $$s_{k+1}=A_{k}s_{k}+w_{k},$$

where *k*=1,2,…,M, *A_k_* ∈R^6×6^ is the matrix that linearly relates the cursor’s kinematics between successive time steps, *w_k_* represents the process noise term, which we assumed to have zero mean and to be normally distributed, i.e. *w_k_* ∼ N(0, *W_k_*), *W_k_*∈R^6×6^, and *M* is the total number of time steps.

Due to subjects following the cursor with their body as if they were controlling it with their shoulders during the calibration phase, we assumed the body motion *observation* to be linearly related to the *state* at each point in time via the stochastic linear function

(4) $$z_{k}=H_{k}s_{k}+q_{k},$$

where *z_k_*∈*R*^*C*×1^ is the vector containing the IMUs’ *observation* at each time step *k. C* is the dimension of the observation vector (24 in this case, but will change for other groups as explained in 3.2.3. *Familiarization*). *H_k_*∈*R*^*C*×6^ is the coefficient matrix that linearly relates the cursor’s state to the body motion, and *q_k_* is the measurement noise term, i.e. *q_k_∼N*(0,*Q_k_*), Q_k_∈R^*C*×*C*^.

In principle, *A_k_, H_k_, W_k_*, and *Q_k_* might change at each time step *k*. However, we made the common simplifying assumptions that they remain constant. Therefore, we can estimate each matrix from calibration data using least squares (for details, see (Wu *et al* 2002)). After the model’s parameters were estimated, the cursor’s kinematics and body motion were now encoded by equation (3) and equation (4) respectively, and subjects could now control a cursor by moving their shoulders.

#### 2.2.3. Familiarization

After the calibration phase, subjects were randomly assigned to one of three groups to perform the rest of the experiment, so that each group had eight subjects. Each group had a different amount of information in their *observation* vector used to control the cursor. Subjects in the first group had only Euler angles in the observation vector (group E), subjects in the second group had Euler angles, and angular velocities (group EV), and subjects in the third group used Euler angles, angular velocities, and linear accelerations to control the cursor (group EVA). Subjects were then allowed to try the mapping in a familiarization phase for 1 min.

They were asked to move through their entire range of motion during the calibration phase. However, performing these types of movements for the whole duration of the experiment would cause exhaustion. Therefore, we reduced the effective range of motion by amplifying the measured motion signals by 300% for the rest of the experiment. This meant that subjects would have to elevate their right shoulder to 33% of their range in order to reach a target located 5 cm above the origin.

Typical joysticks have a mass spring damper system that allows them to come back to the resting position when no force is applied, so we implemented a filter to obtain a similar behaviour. The cursor’s position, or the *x* and *y* in the state, were modelled as forces acting orthogonally on a mass spring damper system described by the equation of motion

(5) $$u_{k}^{\prime \prime }+\frac{b}{m}u_{k}^{\prime }+\frac{k}{m}u_{k}=0,$$

where $u_{k}={\left[x\prime ,y\prime \right]}_{k}^{T}$ represents the cursor’s new, filtered position coordinates. Values for the mass, spring, and damper coefficients were tuned so that the system had a resulting damping ratio of

(6) $$\zeta =\frac{b}{2\sqrt{\text{mk}}}=0.1,$$

There was no specific goal for the familiarization phase, but the subjects were told to try to move the cursor up, down, left, and right several times, to check that they had control of its movements and check that they could bring the cursor back to the origin, and finally, to make sure that they could reach the four corners of the screen. The calibration procedure was repeated if a subject was not comfortable with the map.

#### 2.2.4. Four-target reaching task

Twenty-four subjects performed the first reaching task. Once subjects familiarized themselves with the map, they performed five blocks of a centre-out reaching task. Subjects controlled a blue, 1 cm diameter cursor to reach four targets 4 cm in diameter appearing in random order on the screen 5 cm below, above, to the right, or to the left of the origin. While this study was limited for practical purposes to a few reaching targets, the interface, after the calibration phase, allowed them to move in all directions and to reach all points of the computer display. Subjects were allowed a total of 6 s to complete the task before the target disappeared. Subjects had to remain inside the 4 cm diameter origin target for 200 ms for a new yellow target to appear. The subjects were instructed to reach the targets as quickly and accurately as possible and remain inside them for 1 s. The targets turned green while the cursor was inside them and turned red after the 6 s ‘deadline’, where the trial was logged as a failed attempt and the target returned to the origin.

Subjects performed 24 trials per block, with random target order comprised of exactly six trials in each direction. The experiment consisted of five blocks and there was a 1 min resting period between blocks. This protocol allowed us to chart an explicit learning curve for different performance measures for each of the 24 subjects.

#### 2.2.5. Five-target reaching task

A group of four subjects performed a second reaching task using the map that resulted on the best performance for the *Four-Target Reaching Task*. After following the same *Calibration* and *Familiarization* procedures, subjects performed a set of five training blocks of centre-out reaching, with generalization blocks before and after training. The training and generalization task schedule is shown in table 1.

*Training Trials* consisted on subjects controlling a mouse pointer to reach five targets 2.22 cm in diameter appearing in random order on the screen. Unlike the first reaching task where subjects had to move only one shoulder at a time to reach a target, the five target locations for this task required subjects to combine and coordinate shoulder motions in order to reach them. Subjects were allowed a total of 1 s to reach the target before it changed colour. However, contrary to the first reaching task, subjects had unlimited time after the target changed colour to make corrections and complete the trial by remaining inside the target for 1 s. Subjects performed 20 training trials per block, with random target order comprised of exactly five trials in each direction.

*Blind Trials* occurred in random order within the same block as the *Training Trials* in order to test if subjects formed an inverse model of the shoulder-to-cursor map, or if they were relying purely on visual feedback to control the cursor. The blind trials were to the same locations as the training trials, except that cursor feedback was removed for the first second of the trial. Subjects performed five blind trials per block, with random target order comprised of exactly one trial in each direction.

*Generalization Trials* occurred before and after the five blocks of training. These trials consisted on five additional targets that were not seen during training. The targets were a rotated and scaled version of the training targets (figure 7, bottom row). Target distances were scaled down by 75% in order to ensure that subjects would be able to reach them. The different target locations required subjects to make different combinations of shoulder motions than the ones used during *Calibration* and the ones used during training. In order to prevent a training effect, subjects performed ten generalization trials per block, with random target order comprised of only two trials in each direction.

#### 2.2.6. Virtual navigation task

After completing all blocks the *Five-Target Reaching Task*, subjects were placed in a virtual environment developed by our laboratory using a commercial- grade, 3D gaming engine (Unreal Development Kit, Epic Games, USA). Controlling a virtual wheelchair in the simulated environment provided a safe environment where participants could learn and practice simulated driving tasks without the risks of collisions or serious accidents. The simulator was adapted to use the 2D output of the body–machine interface as the virtual wheelchair’s joystick input. The custom environment reproduced a series of task features that mirrored those that the participant would need to perform in a real wheelchair. These tasks were a modified version of the Wheelchair Skills Test (version 4.1, http://www.wheelchairskillsprogram.ca).

As participants drove the virtual wheelchair along the environment, instructions appeared on the screen telling them where to go and what to do. A research assistant also provided feedback and guided the participant through the tasks. All subjects completed seven tasks one time. The tasks were done in the following order for all participants:

(1) Driving forward in a straight line, turning 90° counterclockwise, driving in a straight line, turning 90° clockwise, driving forward in a straight line, opening a door by pressing a proximity switch, and entering the doorway before the door closed after 10 s.
(2) Parallel parking between two wheelchairs and driving through an open doorway.
(3) Driving forward in a straight line, turning 90° clockwise, driving in a straight line, turning 90° counter-clockwise, driving forward in a straight line, opening a door by pressing a proximity switch, and entering the doorway before the door closed.
(4) Driving in slalom form between a set of three barrels and driving through an open doorway.
(5) Driving forward in a straight line, turning 90° clockwise, driving in a straight line, turning 90° counter-clockwise, driving forward in a straight line, opening a door by pressing a proximity switch, and entering the doorway before the door closed.
(6) Driving in a circle around a barrel with seven outside barrels as a barrier and driving through an open doorway.
(7) Driving forward in a straight line, turning 90° counterclockwise, driving in a straight line, turning 90° clockwise and driving forward in a straight line.

### 2.3. Analysis

Subject performance for each trial was quantified by four different measures.

#### 2.3.1. Performance measures

*Error Frequency* was defined, for each block, as the ratio of failed attempts to total allowed attempts (24 per block) i.e. a ratio of 0.8 would indicate that 80% of the trials in one block were not successfully completed. This measure indicated overall performance.

*Movement Time* was computed as the time between a target appearing on the screen and the target disappearing after 1 s of the subject being inside it (successfully completing the task). This measure indicates the speed with which a subject was able to complete the task.

*Movement Variability* was computed as the standard deviation along the axis orthogonal to the direction of the target. This was a measure of the extent to which the sample points lay in a straight line along that axis.

*Path Length Ratio* was defined as the sum of the Euclidian distance between time consecutive cursor points along the path of one trial, divided over the ideal distance for that trial. This measure indicated the ‘straightness’ or ‘effectiveness’ of the movement. A path length ratio equal to one would indicate that the subject moved ideally from the origin to the target.

All performance measures were averaged over all trials to obtain four values per block (one for each reaching direction) for each subject. This resulted in a total of twenty values per subject for the whole experiment. Together, these performance measures allowed us to elicit differences in the cursor’s path control within each subject, within a group, and between the three different groups. Other performance measures (average distance to target, average movement perpendicular error, maximum perpendicular error, dimen-sionless jerk) were also computed, but they were highly correlated to these four, so these four were enough to characterize movement and performance.

#### 2.3.2. Statistics

A two-way mixed model analysis of variance (ANOVA) was performed on each performance measure with BLOCK (1–5) as the within participant factor and GROUP (E, EV, EVA) as the between-participant factor. Violations of sphericity were corrected by the Greenhouse-Geisser method. A post-hoc comparison using a Tukey correction was performed to test the null-hypothesis that the mean *between* groups at each block was the same. In order to determine if subjects from one group were better than subjects from another group after the five blocks, a post-hoc pairwise comparison using Bonferroni correction was performed to test the null-hypothesis that the mean *between* two groups at the fifth block was the same. These tests were repeated for each performance measure and allowed us to reject the null hypothesis at each block at *p* < 0.05.

A paired *t*-test was used to analyse a group’s overall improvement in performance. There was an average performance for each subject on the first and on the last blocks. We tested the null hypothesis that the mean difference between paired observations of the two blocks was zero. We repeated this test for each performance measure and it allowed us to reject the null hypothesis at *p* < 0.05.

#### 2.3.3. Calibration time

We asked the question of how much calibration time is necessary for our subjects to perform well enough. Ideally, we could calibrate the Kalman filter by having the subject move only once in each direction (or 16 s). In reality, subjects might need to move more than once in each direction in order to perform as well as if they moved the six times to each direction (or 96 s) as in the *Calibration* phase. We repeated the calibration of the filter (learning the mapping matrices) by using six different calibration-phase durations: 16, 32, 48, 64, 80, or 96 s (note that it takes 16 s for the subject to move once to each of the four directions).

We tested each of the six maps on a testing set that consisted of the body-movement *observations* for the last 16 s of calibration data. We ‘fed’ those *observations* into the maps in order to make a prediction of the *state*, and we called this the reconstructed state. We then computed the correlation between the reconstructed *state* and the actual *state*. There was one correlation coefficient for each of the dimensions of the *state (x, y, v_x_, v_y_, a_x_, a_y_)*. This allowed us to test the performance of the calibration for each of the six calibration times.

## 3. Results

### 3.1. Four-target reaching test

**Figure 2:**
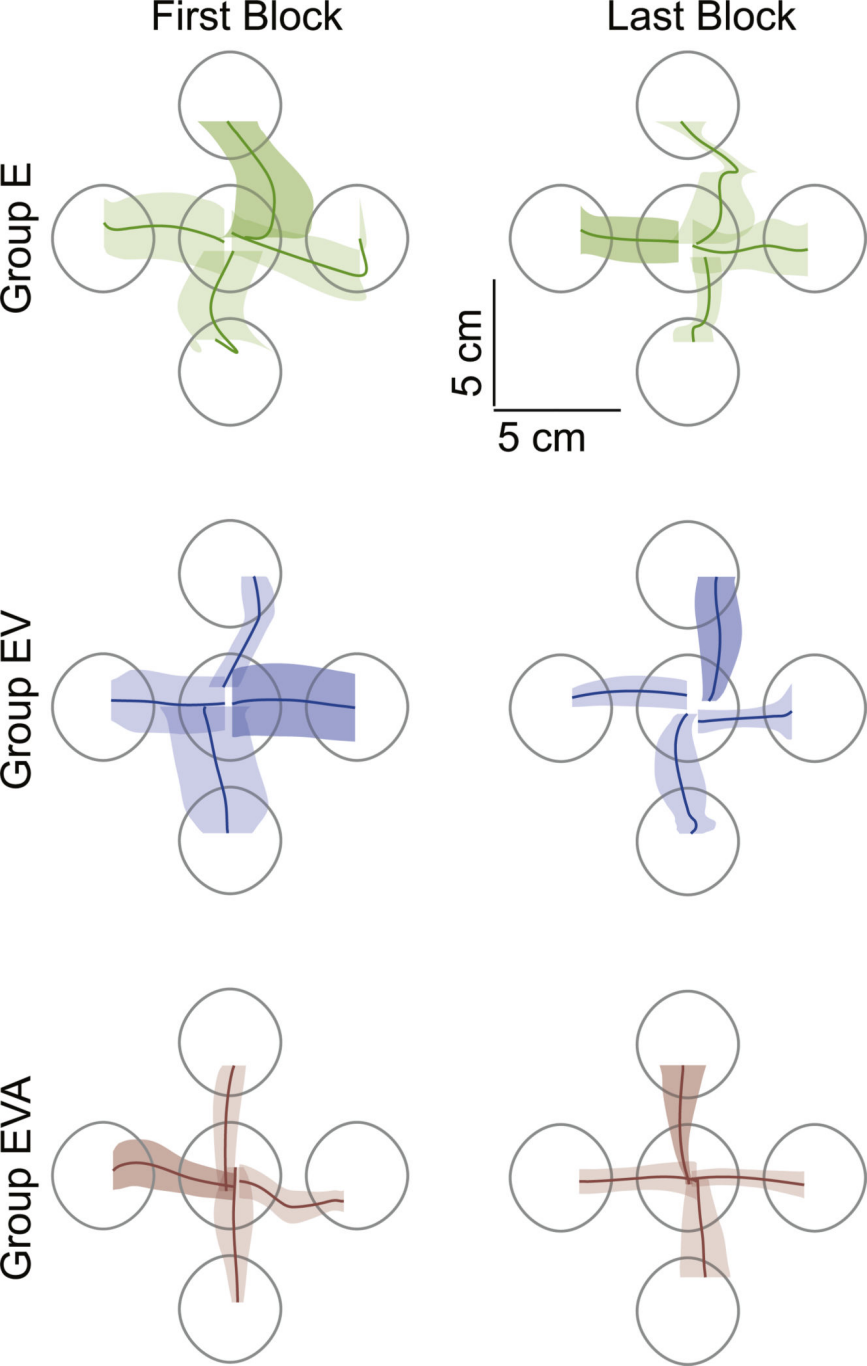
Average movement performance. Movements for representative subjects in each group on their first (left) and last (right) blocks. The dark lines represent the mean for all trials in the same direction for that block, and the shaded area shows the standard error. The grey circles are the 4 cm diameter targets that subjects had to reach to.

All subjects were able to successfully perform the reaching task. Reaching movements in the initial and final phases of training are reported in figure 2. A subject with typical performance for his group was chosen to represent each group in the figure. Movements are represented by the mean paths (dark lines) and their corresponding standard error (shaded area).

Performing the task was somewhat difficult for subjects in the E group for the first block of the reaching test. Trials to each direction were somewhat curved, and the standard error of movements was almost as large as the target diameter. In the following blocks, subjects in the E group continued improving their performance, and by the fifth (last) block their performance was notably better than the first block. The improvement in performance with practice was evident by the mean path becoming straighter and the standard error of the movement becoming smaller.

The task during the first block was not as difficult for subjects on groups EV and EVA. Subjects in both groups were able to move in a straight path in all four directions from the very first block. However, the standard error was larger for subjects on group EV than those on group EVA. Subjects in these groups continued improving their performance, and by the fifth (last) block their performance was noticeably better when compared to the first block. Their path linearity did not change dramatically, but they were able to considerably reduce the variability of their movements as shown by the standard error.

Subjects in all three groups were able to complete the reaching task and improve their control of the two-dimensional (2D) cursor on the screen. Subjects were able to move the cursor in a straight trajectory with their decoded map. The targets were considerably large in diameter, but the standard error of movements was usually smaller than the size of the target. These findings are consistent with the observations that subjects tend to generate rectilinear movements of a visually guided cursor under hand control (Hogan 1984, Flanagan and Rao 1995, Wolpert *et al* 1995).

### 3.2. Performance measures

#### 3.2.1. Five blocks of reaching task

**Figure 3:**
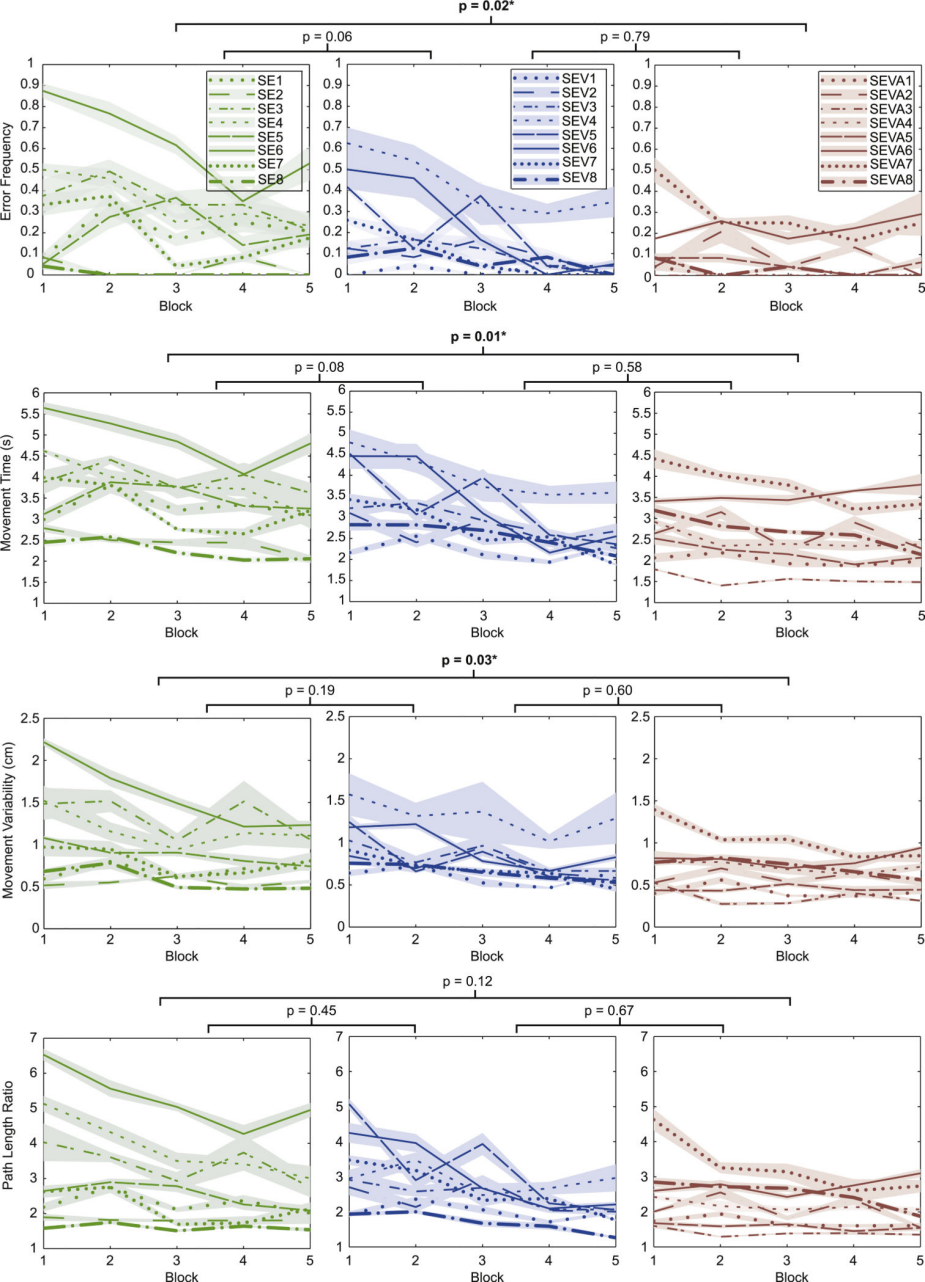
Subject performance for five blocks. Each subject’s mean performance (dark line) and standard error (shaded area) is shown for each of the five reaching blocks. A different line type representing each subject and each group is shown in a different coluor. The results for the ANOVA are shown by the *p*-value on top of the line connecting two plots.

All subjects were able to complete the five blocks of the reaching task. Differences in performance between blocks for the three different groups are shown in figure 3. There was an overall improvement in performance after practice for subjects in the three groups. However, the level of improvement was not consistent across the groups.

*A reduction in error frequency* across the five blocks was apparent for most subjects in all three groups (figure 3, row 1). This indicated that, with practice, subjects became more accurate and were able to control the target well enough to perform the task in the allotted time. There was a block effect on *error frequency* (*p* < 0.01). However, group differences did not depend on block (ANOVA interaction effect block*group p = 0.158). The between-groups difference in *error frequency* was only statistically significant between groups E and EVA, as demonstrated by the results of the Tukey multiple comparison (*p* = 0.016). This value is shown on top of the line connecting both groups’ error frequency panels. Differences in *error frequency* between groups E and EV, and differences between groups EV and EVA were not significant (*p* = 0.063 and *p* = 0.785 respectively). It is important to notice that most subjects on group EVA had an initial *error frequency* of less than 0.1, so even though they reduced their *error frequency* to zero, there was not much change in their performance.

Subjects were allowed a total of 6 s to complete the task before the target disappeared, however subjects in all three groups learned to reach the target in much less time. With practice, the general trend of all three groups seemed to be to reduce the *movement time* (figure 3, row 2). By the fifth block subjects in all three groups had a shorter *movement time* than their first block. This indicated an improvement in the control and familiarization with the 2D cursor using their shoulders. There was a block effect on *movement time* (*p* < 0.01). However, group differences did not depend on block (ANOVA interaction effect block*group p = 0.398). The between-groups difference in *movement time* was statistically significant between groups E and EVA (*p* = 0.011). Differences in *movement time* between groups E and EV, and differences between groups EV and EVA were not significant (*p* = 0.083 and *p* = 0.583 respectively).

A reduction in *movement variability* across the five blocks was also apparent for most subjects in all three groups (figure 3, row III). Subjects on group E had higher *movement variability* than subjects on groups EV and EVA for the first block. This result suggested that subjects learned to decrease the variability of their movements and converged to one movement strategy. Accordingly, they possibly learned to reduce movements that were unnecessary or caused the cursor to move in an undesired direction. There was a block effect on *movement variability* (*p* < 0.01). However, group differences did not depend on block (ANOVA interaction effect block*group *p* = 0.474). The between-groups difference in *movement variability* was only statistically significant between groups E and EVA (*p* = 0.031). Differences in movement variability between groups E and EV, and differences between groups EV and EVA were not significantly different (*p* = 0.191 and *p* = 0.603 respectively).

As mentioned in the *Reaching Test* section, subjects moved towards a straighter trajectory of the controlled cursor. This was quantified by an overall reduction of *path length* across the five blocks of the experiment. There was a block effect on *path length* (*p* < 0.01). However, group differences did not depend on block (ANOVA interaction effect block*group p = 0.220). Interestingly, the between-groups difference in *path length* was not statistically significant between any two groups. The difference between groups E and EVA was not significant (*p* = 0.118). Differences in *path length* between groups E and EV, and differences between groups EV and EVA were also not significant (*p* = 0.447 and *p* = 0.671 respectively).

The results for the post-hoc pairwise comparison at block five can be seen in table 2. Significant differences in performance on the fifth block were observed mostly between groups E and EV and between groups E and EVA. However, there was not significant difference in performance at the fifth trial between groups EV and EVA.

#### 3.2.2. Learning analysis

**Figure 4:**
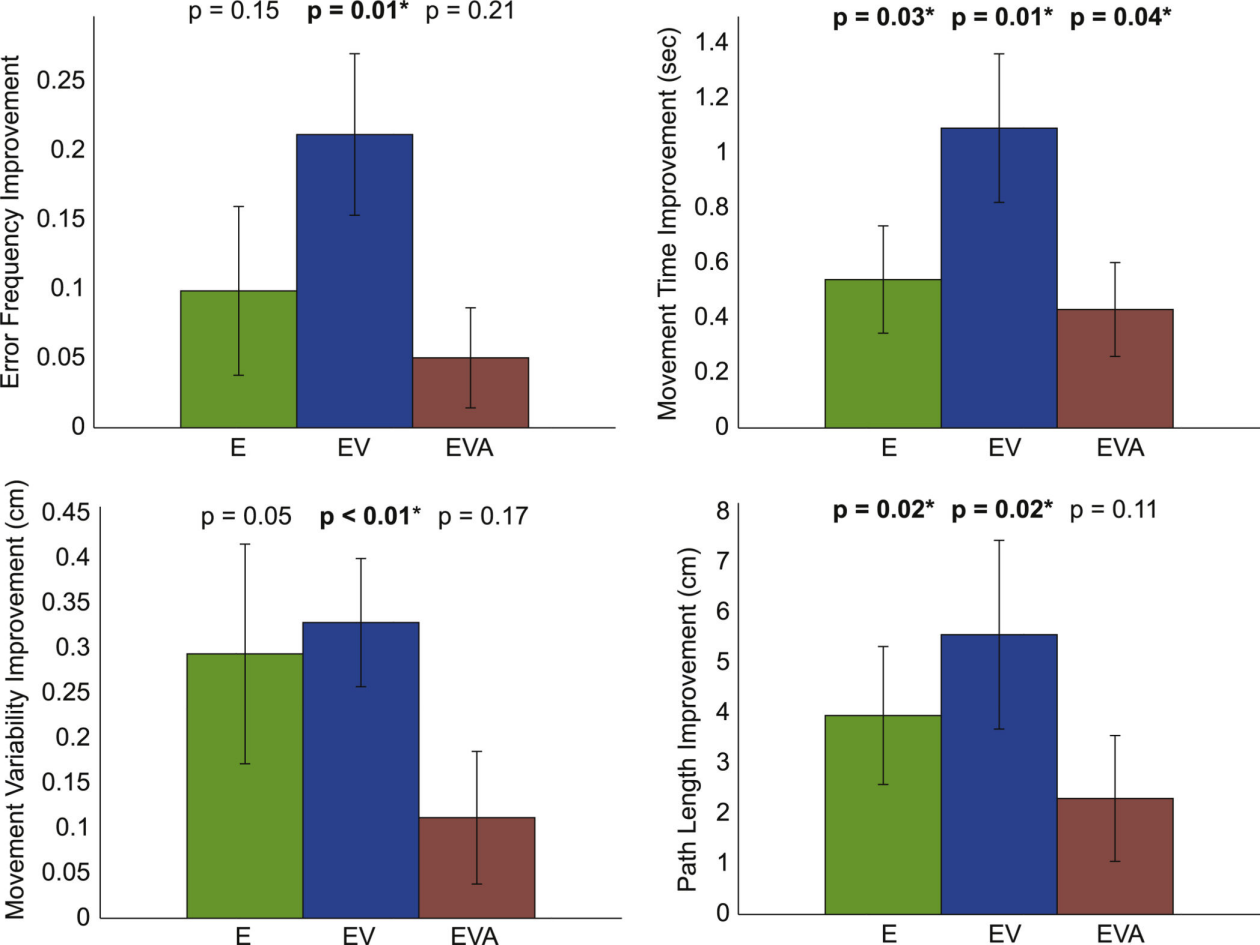
Learning effect. The bars show the mean difference between the first and the last blocks for each group. A positive value indicates an improvement in performance, and a negative value indicates performance degradation. The error bars represent the standard error. The results of the paired *t*-test are shown by the *p*-value on the top of each plot, with an asterisk indicating a significant learning for that group.

Most subjects were able to learn and improve their performance as evaluated by all four measures. Learning averages for each group are shown in figure 4. Each group’s bar represents the mean of the differences between the first and the last block for all subjects in that group. A positive value indicates an improvement in performance. The results of the paired *t*-test are shown by the *p*-value on the top of each plot. An asterisk and bold number indicate a significant learning for that group. The learning effect was not consistent between groups. Groups E and EV seem to show a stronger learning effect than group EVA. This was mostly due to a ceiling effect, because subjects on group EVA might have been at a ceiling of performance since the very first block.

### 3.3. State estimation accuracy depends strongly on calibration time

**Figure 5:**
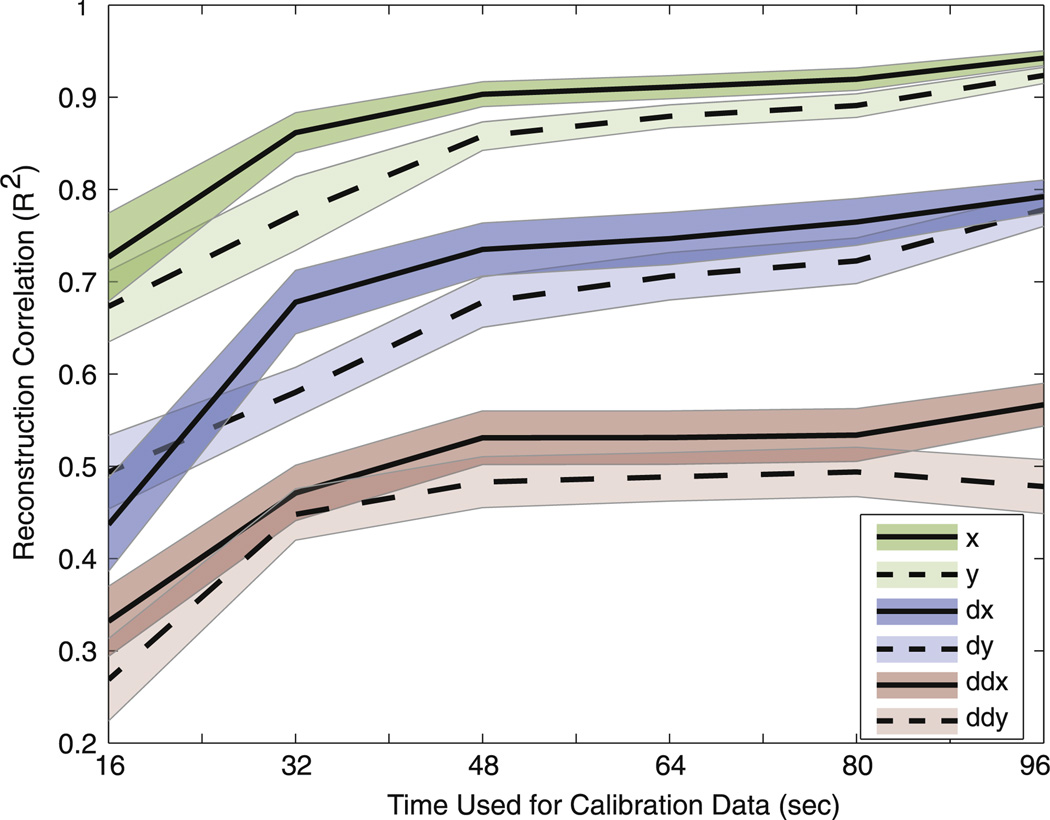
Reconstruction of calibration times. The correlation coefficients for each of the dimensions of the state are shown for each of the six calibration times. The lines represent the mean between all 24 subjects, and the shaded areas indicate the standard error.

The correlation coefficients between the actual *states* and the reconstructed *states*, using different durations of calibration data, are shown in ‘figure 5’. As more data was available (or calibration time increases), the correlation coefficients for position, velocity, and acceleration increase. The reconstruction was always stronger for position than for velocity, and acceleration is the weakest. Even though there was a noticeable difference between training with 16 s and 32 s, there was not much improvement after performing the reconstruction with 48 s or more. These results indicate that subjects could effectively calibrate the filter in only 48 s. This calibration time would be considerably shorter than the current, state of the art, EEG motor imagery methods that take around two hours to calibrate (Carlson and Millán 2012).

### 3.4. Five-target reaching test

**Figure 6:**
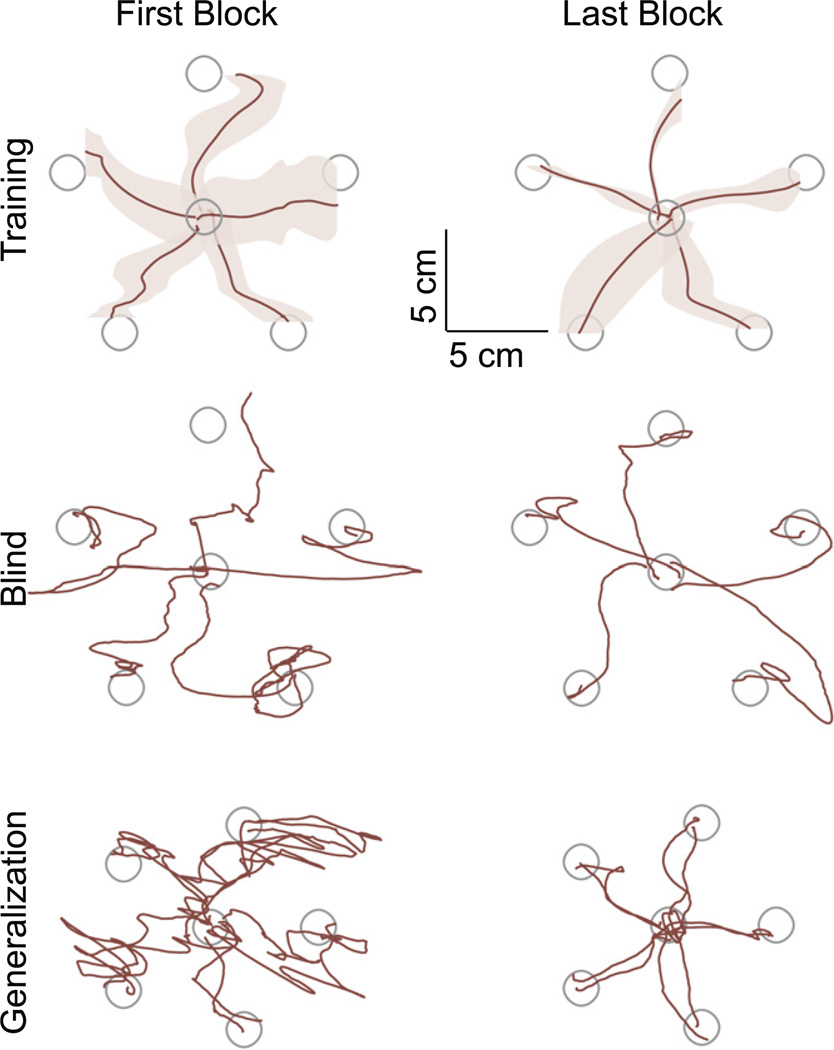
Movement performance for training, blind, and generalization trials. Movements for one subject on the first (left) and last (right) blocks. Top: the dark lines represent the mean for all training trials in the same direction for that block, and the shaded area shows the standard error. Middle: the dark lines represent the raw paths for each target during the blind trials within the first and the last blocks. Bottom: the dark lines represent the raw paths for each target during the generalization blocks. Note that these do not include a standard error shaded area, because there were only two trials per target. The grey circles are the 2.22 cm diameter targets that subjects had to reach to.

All subjects were able to perform successfully the reaching task to five targets on the screen using the EVA map. Reaching movements in the initial and final phases of training for one representative subject are reported in figure 6. Movements are represented by the mean paths (dark lines) and their corresponding standard error (shaded area).

Performing the task was a little more difficult for subjects doing the *Five-Target Reaching Task* than for subjects doing the *Four-Target Reaching Task*. Not only was the target half the size for the *Five-Target Reaching Task*, but also subjects had to coordinate the movement of two shoulders simultaneously in order to reach them. In the first block, trials to each direction were curved and the standard error of movements was almost as large as the target diameter. However, subjects continued improving their performance during the following blocks, and by the fifth (last) block their performance was notably better than the first block. The improvement in performance with practice was evident by the mean path becoming straighter and the standard error of the movements becoming smaller.

Five blind trials (one per target location) were randomly introduced during each block. During blind trials, the visual feedback of the cursor’s location was turned off for the first second of the trial. From the first block, subjects moved in the correct general direction for each target during the first portion of their reach (figure 6, middle row). However, they did not get very close to the exact target location, and they had to make adjustments in position after the cursor’s visual feedback returned in order to get inside the targets and complete the trials. Subjects continued improving their performance during the following blocks, and by the fifth (last) block their performance was notably better than the first block. With practice, subjects were moving closer to a straight line towards each target and they got closer to the exact target location, so fewer adjustments in cursor position were needed after the cursor’s visual feedback returned.

Before and after the five blocks of training, subjects were asked to perform a set of ten generalization trials to five target locations that were not seen during training (figure 6, bottom row). The first generalization block was somewhat difficult for all subjects. Trials to each direction were curved and subjects overshot the target. There were several changes in movement direction, and several adjustments in position were needed in order to complete the task. At this point, subjects were not experienced with the map. The second generalization block, which happened after the five blocks of training, was not difficult for any of the subjects. Subjects were able to move in a much straighter trajectory towards each target, did not overshoot the targets, and needed minor adjustments in cursor position in order to complete the trials.

### 3.5. Virtual navigation task

**Figure 7:**
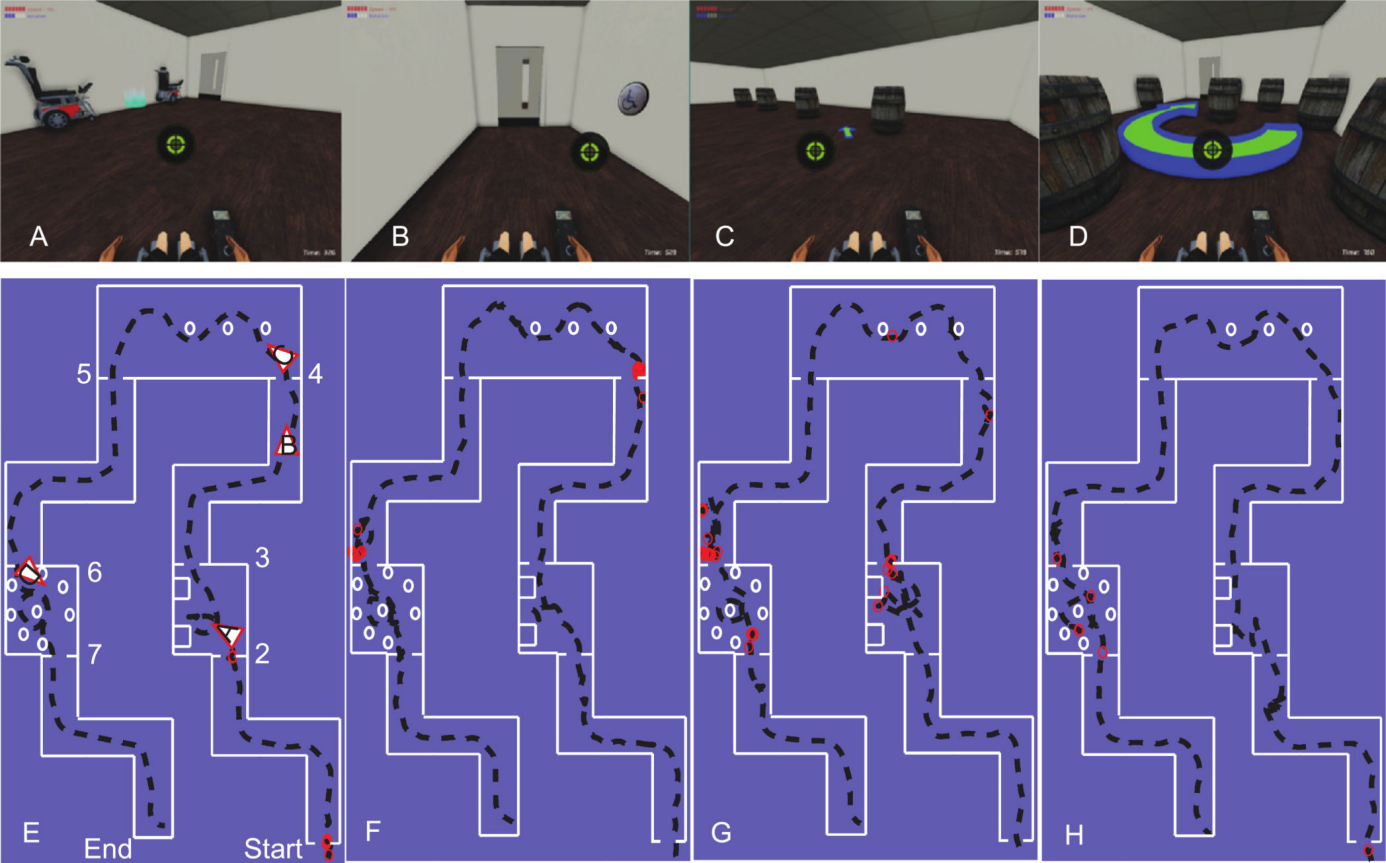
Virtual wheelchair navigation by Kalman filter using a non-invasive body–machine interface. Top: subjects controlled the wheelchair’s joystick position (green circle) in order to move around. Besides navigating the map without hitting the walls, subjects were required to perform tasks such as parallel parking (A), opening doors by pressing the door proximity switch (B), slalom through a set of three barrels (C), and going around in a circle around a barrel (D). Bottom: driving paths in the virtual reality environment are shown for three subjects (E–G). The white lines represent the walls. The black dotted lines represent the subjects’ paths. The triangles indicate the position and orientation of the camera for the pictures on the top. The numbers indicate the starting point for each of the required tasks. The red circles represent collisions between the simulated wheelchair and another object in the virtual reality environment.

All subjects were able to successfully perform the wheelchair navigation task in the virtual environment using the EVA map. Screenshots of the virtual environment and trajectories for all subjects are shown in figure 7. The dark dotted lines represent each subject’s trajectory within the map, and the red circles indicate locations where the subject’s simulated wheelchair collided with another object in the virtual environment.

Subjects’ trajectories were generally smooth and straight during open hallways and 90° turns (figure 7, plot E, tasks 1,3,5, and 7). There were no specific instructions on how to parallel park, besides getting the wheelchair to sit still inside the blue glowing circle in figure 7, plot A. Some subjects went directly forward and stopped there, while others parallel parked with a similar strategy as parking a car. They moved to be parallel with the front wheelchair, and then they backed up into the spot while turning. This was a more difficult strategy, as the camera point of view remained fixed on the back, so they could not see the wheelchair behind them nor the wall on their left.

Opening doors and going through them was the most difficult task for all subjects. Subjects were required to activate a proximity switch by getting close to it (figure 7, plot B). The door took 7 s to open completely, remained opened for 10 s, and then took another 7 s to close. Doors with proximity switches were located at the start, at the end of tasks 1, 3, and 5. This is where most of the collisions happened. Every subject experienced a collision in a proximity switch door at least twice throughout the virtual navigation task.

The 4th task, or the slalom task, was not too difficult for subjects (figure 7, plot C). Subjects were required to navigate through three barrels by moving in an ‘S-like’ path. Only one of the subjects collided against one of the barrels by turning too sharply (figure 7, plot G).

Navigating in a circle around a barrel was not too difficult for subjects (figure 7, plot E). Subjects were required to go around the barrel without touching the barrels on the sides. All subjects were able to go around the barrel smoothly. However, one of the subjects collided against the barrels surrounding it (figure 7, plot H).

Requiring subjects to perform different tasks allowed them to practice in a safe and controlled environment. These results demonstrate the validity of using a body–machine interface with a Kalman filter to control a 2D cursor on a screen and to control a simulated powered wheelchair.

## 4. Discussion

Our results demonstrate that the Kalman filter decoding of upper-body motions is appropriate for assistive device applications requiring 2D control. Experimental results in brain–machine interfaces with this well understood probabilistic approach have shown its superiority to other traditional linear filtering methods (Wu *et al* 2003, Wu *et al* 2004). The Kalman filter does not require long time windows in which to collect data, it is simple to train, and the real-time implementation is trivial. Additionally, the Kalman filter provides a clear statistical interpretation (Welch and Bishop 1995), an explicit generative model, an incremental estimate of the state that improves over time, and an estimate of the uncertainty in the state (Wu *et al* 2002).

Different methods have recently been proposed for people with high tetraplegia to control their powered wheelchairs. EEG methods provide an alternative when all mobility has been lost—as in locked-in syndrome or advanced multiple sclerosis (Iturrate *et al* 2009, Carlson and Millán 2012). However, these methods are computationally expensive, have low bandwidth, require long training and familiarization phases, and demand high concentration from the user. While the rate of information transmission of non-invasive brain computer interfaces ranges from 0.05 to 0.5 bits s^−1^ (Wolpaw *et al* 2000, Townsend *et al* 2010), a recent study estimated that body motions may operate at about 5 bits s^−1^ (Townsend *et al* 2010). IMUs mounted at the back of the head to convert head movements into steering commands can provide a satisfactory alternative when some neck motion remains available (Mandel *et al* 2007). However, interactions are limited to the head only and fail to promote upper-body coordination when users might still have significant residual motion capability.

Calibration time is an important factor that influences whether people adopt a technology or not. Current brain–-machine interfaces take from 4 min in ECoG to a couple hours in EEG to properly calibrate. We investigated what would be the minimum calibration time that subjects could work with, without negatively affecting the performance of the decoding. As expected, the reconstruction of the testing set improved as the duration of the calibration set increased. There was a noticeable difference between calibrating with 16, 32, or 48 s. However, there was not much improvement in the reconstruction after calibrating with more than 48 s of data. It is important to note that the speed of the moving cursor during the calibration was relatively slow, and it took 16 s for the cursor to move once to each of the four directions. It is possible that increasing the speed of the cursor could decrease the calibration time further.

Subjects were able to generalize to targets that were a rotated and scaled-down version of the ones presented during training. These targets required subjects to make different combinations of shoulder motions. Even though the generalization targets were a scaled-down version of the training targets, subjects did not overshoot them. Moreover, subjects were able to perform the reaching trials even when the visual feedback of the cursor was removed. These results suggest that, after practice, subjects had a great understanding on how the magnitude of their shoulder movements affected the magnitude of the cursor movements. Our results are in agreement with studies suggesting the formation of an internal model between body and cursor motions (Wolpert *et al* 1998, Todorov and Jordan 2002, Emken *et al* 2007, Berniker and Kording 2008, Casadio *et al* 2010).

All four subjects were able to navigate the virtual environment in the simulated wheelchair after training in a centre-out reaching task. These results demonstrate the feasibility of the body-machine interface using IMUs on the shoulder to control a virtual wheelchair. Earlier studies have demonstrated that the skills acquired while practicing the control of a virtual wheelchair are at least partially retained and ‘generalized’ when controlling an electrically powered wheelchair (Cooper *et al* 2002, Holden 2005). However, it is important to note that subjects did experience collisions between the simulated wheelchair and other objects in the environment. This highlights the importance of practice until subjects reach a high level of performance before they drive a real wheelchair. It is important to minimize the likelihood of accidents and collisions while driving an electrically powered wheelchair.

### 4.1. Clinical implications

Subjects in all groups were able to learn to control the cursor using IMUs on their shoulders. They all successfully completed the reaching task, but performance varied between the groups. Subjects in the EVA group outperformed subjects in the EV and E groups in all of the performance measures. Our findings confirm the ability of the motor control system to exploit motor redundancy for reorganizing motor coordination (Chen *et al* 1998, Gréa *et al* 2000, Casadio *et al* 2010, Casadio *et al* 2011). However, a significant learning effect was not observed for subjects on group EVA. Learning happened mostly for groups E and EV. These results might suggest that subjects who use all available body kinematics information may reach a ceiling in performance early in the experiment.

We analysed the effect that adding more kinematic information in the observation vector of the decoding algorithm had on cursor control performance. Adding too much information in the procedure could add unwanted noise and be detrimental to the control of the cursor. Our results demonstrate that this was not the case, and they suggest that algorithms with greater redundancy (and perhaps more sensors) are desired for BMI decoding.

Having the ability to monitor motor function is highly desirable in order to automate adaptation of therapy based on patients’ needs and improvements (Krebs *et al* 2003, Reinkensmeyer *et al* 2004, Choi *et al* 2009, Li *et al* 2009, Kan *et al* 2011). Adaptive online algorithms have been shown to be useful in many scenarios including motor control (Mak and Wolpaw 2009, Townsend *et al* 2010, Cunningham *et al* 2011). Our system also allows adapting and updating our models with data collected during subject-controlled trials, instead of keeping them fixed after the calibration phase.

Although not reported in detail here, the number of ‘re-calibrations’ needed during the course of the experiment was noticeably different for each group. All subjects moved and changed their posture during the length of the experiment. This caused the sensors to have a different resting (or zero) reading, and the subjects’ resting position caused the cursor to drift from the origin of the screen. We asked subjects to pay attention to this detail and let us know when they felt their resting position was no longer in the origin. At these occurrences, we ‘zeroed’ the signals so that their current position was in the origin. This was a recurring problem (about one ‘re-calibration’ per block) for subjects on group E, but was not a common occurrence (about one ‘re-calibration for the complete session) for subjects on group EVA. Subjects on group E were only using the angles of the sensors to move their shoulders, so changes in posture had a noticeable effect on the origin. When angular velocities and linear accelerations were used for the map, maybe their posture had changed, but their instantaneous velocities and accelerations were not changing and thus the cursor had a greater tendency to remain close to the origin. In more recent experiments, we have subjects performing blocks on different days. We mark the position of the sensors on the vest for each subject and we place the sensors accordingly. Subjects are able to perform the reaching task from the first trial. We envision users of our system getting up in the morning, putting on the vest with the IMUs in the marked position, ‘zeroing’ the signals once, and go through their day with the same map.

### 4.2. Limitations

Establishing the optimal sensor kinematics of the decoding algorithm was the main focus of this study. In order to reduce variability introduced by different body-to-cursor maps, the design of our experiment instructed specific movements in the calibration phase for all subjects. In the application of this BMI, subjects will have the ability to choose their own movements to control each of the directions of the cursor. Although some movements will be easier for some subjects than for others, we expect our observations of taking advantage of redundancy to carry over regardless of the chosen map.

This study demonstrated promising results on 2D cursor and virtual wheelchair control by a BMI on unimpaired subjects. However, one should not simply assume these findings could be extrapolated with people with paralysis. People with poor control of their body might not be able to control the velocity and acceleration of their movements as smoothly as unimpaired subjects. Our next step is to conduct experiments on spinal cord injured participants to confirm our observations.

## 5. Conclusion

This study provides us with the platform for people with higher spinal cord injury to control a virtual cursor and a virtual wheelchair. The use of IMUs on the shoulder area can potentially replace the current sip-and-puff and head-and-chin systems for people with some motor and sensory capacity remaining on the upper-body. The control of the cursor can be easily turned into the control of a joystick that in turn controls a powered wheelchair. This type of control would allow for an interface that is not obstructive to the head or the mouth and has a proportional, continuous directionality control. This body–machine interface is able to adapt to each individual’s residual mobility, and could keep evolving together with the user’s skills while promoting learning through upper-body coordination. With successful training, users could improve their independence by enhancing their movement capabilities that survived the injury.

## Figures and Tables

**Table 1:** Training and generalization task schedule.

Block #	Gen 1	Block 1		Block 2		Block 3		Block 4		Block 5		Gen 2
					
Phase	Gen	Train	Blind	Train	Blind	Train	Blind	Train	Blind	Train	Blind	Gen
Number of trials	10	20	5	20	5	20	5	20	5	20	5	10
Visual feedback	Y	Y	N	Y	N	Y	N	Y	N	Y	N	Y

**Table 2:** Group pairwise comparisons at block five.

Measure	(I) Group	(J) Group	Two sample *t*-test significance (Bonferroni)
	E	EV	0.01*
Error frequency	E	EVA	0.02*
	EV	EVA	1.00
	E	EV	0.02*
Movement time	E	EVA	0.01*
	EV	EVA	1.00
	E	EV	0.04*
Movement Variability	E	EVA	0.01*
	EV	EVA	1.00
	E	EV	0.17
Path length	E	EVA	0.11
	EV	EVA	1.00
